# Dr. Per-Ingvar Branemark: The Father of Modern Dental Implantology

**DOI:** 10.7759/cureus.73950

**Published:** 2024-11-18

**Authors:** Pragati Rawat, Deepesh Saxena, Abhinav Sharma

**Affiliations:** 1 Prosthodontics and Crown and Bridge, Subharti Dental College and Hospital, Swami Vivekanand Subharti University, Meerut, IND; 2 Oral Medicine and Radiology, Subharti Dental College and Hospital, Swami Vivekanand Subharti University, Meerut, IND

**Keywords:** bone anchoring hearing aid, brånemark, dental implants, dental rehabilitation, historical vignette, osseointegration, prosthetic limbs

## Abstract

Swedish physician and anatomist Professor Per-Ingvar Brånemark (1929-2014), often known as Sir Brånemark, discovered the osseointegration phenomenon that revolutionized the dental implantology field. Even though he was an orthopedic surgeon, Sir Brånemark's groundbreaking contributions to dentistry in the form of the Brånemark System of dental implants established the groundwork for modern fixed prosthodontics. The dental community still values his efforts today. His influence has not only been seen in the realm of dentistry but also in medicine, where the phenomenon of osseointegration has led to the development of prosthetic limbs as well as the development of cochlear implants. Based on a thorough literature search conducted across many databases, including PubMed and Google Scholar, this study offers a scientific overview of Sir Brånemark's contribution to implantology. His legacy lives on through the Brånemark Osseointegration Centre (BOC), founded in 1989 in Gothenburg, Sweden.

## Introduction and background

**Figure 1:**
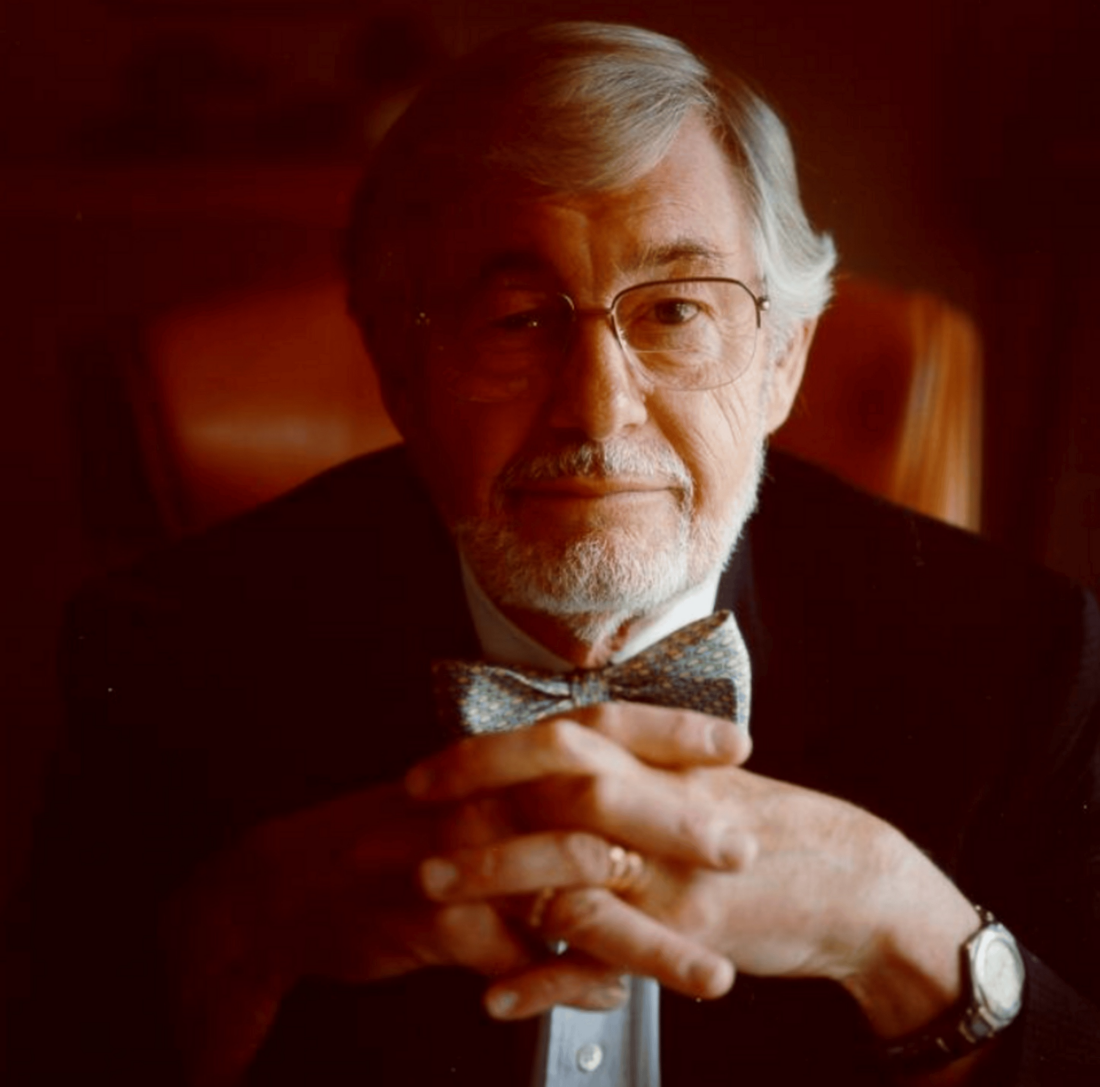
Sir PI Brånemark Source: Wikipedia [3]; Released for free-use by Brånemark himself; CC BY-SA 3.0

Sir Brånemark's (Figure 1) contribution to the field of dental implantology in the form of osteointegration is well-known. Beginning in Gothenburg in 1965, he researched dental implants. In 1982, he presented his groundbreaking discoveries at the historic Toronto Conference. He introduced the idea of the new technology of machined titanium implants to both North America and the rest of the globe, describing the durability of the living bones's biomechanical and biological fusion with titanium. It was then that Bofors (now Nobel Biocare) began commercializing the Brånemark Implant System and treatment approach, making it accessible to patients worldwide for the replacement of lost teeth [1,2].

This review delves into Brånemark’s accomplishments by scrutinizing his research, methods, and the influence of his work on contemporary dentistry as well as medical practice.

## Review

Early life and career

Professor PI Brånemark was originally known as Per Ingvar Persson, and being an anatomist by profession, he published various articles related to the same subject. It was later in the 1950s that he changed his name from ‘Persson’ to ‘Brånemark’ meaning ‘who breaks ground', and today his research work on titanium and dental implants has actually shown groundbreaking results. He completed his studies at the University of Lund, Sweden, in 1959 and soon after became a professor of anatomy at Gothenburg University, Sweden, in 1969 [1,3].

Contribution to dentistry

Although the research on dental implants by Sir Brånemark began in 1965, its foundation was laid way earlier, in the 1950s, when Sir Brånemark discovered the potential of titanium implants [4]. He found that titanium can be fused to living bone and that the two cannot be separated unless fractured. He called this phenomenon 'osseointegration’ and this term holds the utmost importance in the field of rehabilitation, be it orthopedics or dental science to date [5].

He discovered osseointegration in the early 1950s, but financial support in the form of a grant from the US National Institutes of Health (NIH) did not come his way until years later and after several denials [6].

**Figure 2:**
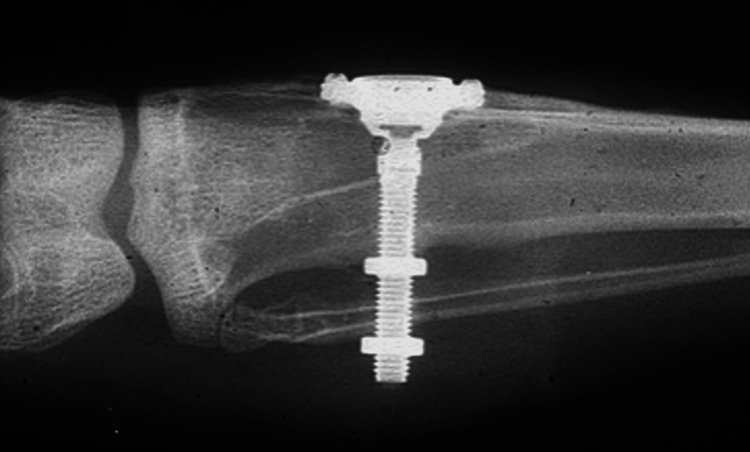
Radiograph of Brånemark's first implant surgery (implant placed in the rabbit’s tibia and fibula) Source: Wikipedia [3]; CC BY-SA 3.0

A series of in vivo studies on bone, marrow, and joint tissue were conducted after Sir Brånemark's earlier study on rabbit’s fibula revealed a close functional connection between marrow and bone in the repair of bone defects to assess the mechanical, chemical, thermal, and rheological reaction of the injured tissue. Consequently, long-term in vivo microscopic examinations of the bone and marrow reactions to screw-shaped titanium chamber implants in rabbit tibia and fibula were conducted. (Figure 2) [7,8]. This was shortly followed by the placement of screw-shaped titanium chambers in the dental extraction sockets in dogs, which were later loaded with fixed prostheses after three to four months of healing. The span of such osseointegrated implants was estimated to be up to 10 years. Also, the lack of contamination and the inseparable bond between titanium and bone gave way to reconstructive surgeries for repairing significant mandibular abnormalities in dogs using osseointegrated implants in conjunction with autologous bone grafts. It was observed that the success of osseointegrated implants was attributed to the fact that during the healing period, a shell of compact cortical bone was formed around the implant without any intervening soft tissue between the implant’s surface and the living bone. [5,8].

After experimenting with animals, Sir Brånemark and his team moved on to human experiments, where they placed an optical titanium chamber in the left upper arm of 20 healthy volunteers [6, 8]. After conducting crucial microscopic studies on these subjects to examine blood cell intravascular behavior and microcirculation at high resolution, they found no evidence of inflammation and concluded that bone anchorage based on the osseointegration principle might also be effective in humans [8]. And, finally, in 1965, Gösta Larsson received four mandibular implants for a complete arch-fixed implant-supported metal-resin prosthesis, which lasted for his entire lifetime, i.e., 40 years [1]. Brånemark's dental implants were subsequently authorized by Sweden's National Board of Health and Welfare in 1970. Furthermore, the Brånemark System is still marketed and sold by the biomedical firm he founded in Sweden in 1981 [6].

Contribution to the field of otology

Anders Tjellström, a PhD candidate and part of Brånemark's research team, adopted Brånemark's notion that a titanium implant in the bone behind the ear may function as a type of hearing aid. In the mid-1970s, Sir Brånemark and Tjellström subsequently performed the first implant procedure, which involved reconnecting an Oticon bone vibrator to a snap coupling attached to a dental implant and linking it to an audiometer. This restored the patient's hearing after 30 years of hearing loss. It was observed that the sound propagated through the bones of the maxilla to the inner ear, resulting in a very high, clear sound [6,9]. This eventually resulted in the production of these cochlear implants, known as the Bone Anchoring Hearing Aid (BAHA) system in 1999, which began selling in 2005 under the name Cochlear Bone Anchored Solutions [9]. Since then, over 1,00,000 patients have benefitted from BAHA devices.

Contribution to the field of orthopedics

Significant advancements in orthopedics have resulted from Sir Per-Ingvar Brånemark's groundbreaking study on osseointegration, which was first designed for dental implants. Thanks to his efforts, novel orthopedic implants that fuse with bone have been created, making major improvements in joint replacement and prosthetic technology possible.

Torgny Haraldson discovered in 1979 that the presence of positive sensory input in osseointegrated bridge patients resulted in masticatory performance that was equivalent to the native dentition, establishing the notion that osseoperception is a component of osseointegration [10]. With this idea in mind, as well as the osseointegration theory put forward by Sir Brånemark, Rickard Brånemark and his father, PI Brånemark, created orthopedic prostheses (prosthetic limbs and legs) attached to the human skeleton [3]. Sir Brånemark's work also extended to joint replacements, such as hip and knee prostheses, where osseointegration techniques have been employed to enhance the stability and longevity of these implants [11, 12]. Since then, more than 200 patients have benefited from such an artificial prosthesis.

Awards and honours

Some of the well-known prestigious awards and honors received by Sir Brånemark are presented in Table 1 in chronological order. Apart from these, Sir Brånemark also received the Harvard School of Dental Medicine Medal in the United States for his exceptional contribution to the field of implantology. Additionally, he was the recipient of over 30 honorary posts in Europe and North America, including one from the Royal Society of Medicine in the United Kingdom [1,3].

**Table 1:** Sir Brånemark's achievements

S. No	Year of receiving awards/honours	Awards/honours received	Awarding agency
1	1992	Söderberg Prize (Mini Nobel)	Swedish Society of Medicine
2	1992	Prestigious Medal for Technical Innovation	Swedish Engineering Academy
3	2003	Honorary Doctorate	European University of Madrid
4	2011	Lifetime Achievement Award	European Inventor Award
5	2012	Lifetime Achievement Award	The Greater New York Academy of Prosthodontics

## Conclusions

Sir Brånemark's remarkable journey has ignited many young minds, and innovations in the field of dentistry continue. He died at the age of 85 in 2020. His unspeakable contribution to the field of dental implantology as well as orthopedics and otology has helped many patients. He has truly lived up to what he once quoted: “No one should have to die with their teeth in a glass of water beside their bed.” His work has been a source of inspiration for many implantologists all over the world. Although he was an orthopedic surgeon and not a dentist, his contribution to dentistry can never be forgotten.
